# 6-Hydr­oxy-7-isopropyl-1,1,4a-trimethyl-2,3,4,4a,10,10a-hexa­hydro­phenanthren-9(1*H*)-one

**DOI:** 10.1107/S1600536808007769

**Published:** 2008-03-29

**Authors:** Nezha Rajouani, My Youssef Ait Itto, Ahmed Benharref, Aziz Auhmani, Jean-Claude Daran

**Affiliations:** a Laboratoire de Chimie Biomoléculaire, Substances Naturelles et Réactivité, Université Cadi Ayyad, Faculté des Sciences Semlalia, BP 2390 Marrakech, Morocco; b Laboratoire de Chimie de Coordination, UPR-CNRS 8241, 205 route de Narbonne, 31077 Toulouse cédex, France

## Abstract

The title compound, C_20_H_28_O_2_, commonly named Sugiol, is a natural oxygenated diterpene that we have isolated for the first time from a hexane extract of the fruits of *Juniperus Oxycedrus L.* Its X-ray crystal structure determination confirms an abietane skeleton which was predicted by spectroscopic analysis, mainly by ^1^H and ^13^C NMR. The cyclo­hexane ring adopts a flattened chair conformation, while the cyclo­hexene ring adopts an envelope conformation. The mol­ecules are linked through O—H⋯O hydrogen bonds to form a zigzag chain extending parallel to the *c* axis.

## Related literature

For related literature, see: Bai-Ping & Isao (1991 ▶); Bouhlal *et al.* (1988 ▶); Cremer & Pople (1975 ▶); Iwamato *et al.* (2003 ▶); Politi *et al.* (2003 ▶); Ulubelen *et al.* (1997 ▶).
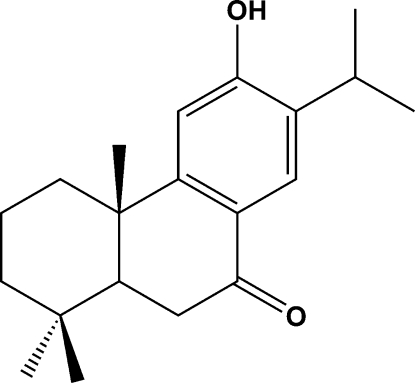


## Experimental

### 

#### Crystal data

C_20_H_28_O_2_
*M* *_r_* = 300.42
Orthorhombic, 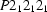

*a* = 9.6060 (4) Å
*b* = 12.6617 (6) Å
*c* = 14.0920 (7) Å
*V* = 1713.99 (14) Å^3^
*Z* = 4
Mo *K*α radiation
μ = 0.07 mm^−1^
*T* = 180 (2) K
0.31 × 0.16 × 0.07 mm

#### Data collection

Oxford Diffraction Xcalibur diffractometer
Absorption correction: none
13398 measured reflections
2003 independent reflections
1212 reflections with *I* > 2σ(*I*)
*R*_int_ = 0.068

#### Refinement

*R*[*F* ^2^ > 2σ(*F* ^2^)] = 0.046
*wR*(*F* ^2^) = 0.154
*S* = 1.05
2003 reflections
205 parameters
H-atom parameters constrained
Δρ_max_ = 0.36 e Å^−3^
Δρ_min_ = −0.38 e Å^−3^

### 

Data collection: *CrysAlis CCD* (Oxford Diffraction, 2007 ▶); cell refinement: *CrysAlis RED* (Oxford Diffraction, 2007 ▶); data reduction: *CrysAlis RED*; program(s) used to solve structure: *SIR97* (Altomare *et al.*, 1999 ▶); program(s) used to refine structure: *SHELXL97* (Sheldrick, 2008 ▶); molecular graphics: *ORTEPIII* (Burnett & Johnson, 1996 ▶), *ORTEP-3 for Windows* (Farrugia, 1997 ▶) and *PLATON* (Spek, 2003 ▶); software used to prepare material for publication: *WinGX* (Farrugia, 1999 ▶).

## Supplementary Material

Crystal structure: contains datablocks I, global. DOI: 10.1107/S1600536808007769/pk2088sup1.cif

Structure factors: contains datablocks I. DOI: 10.1107/S1600536808007769/pk2088Isup2.hkl

Additional supplementary materials:  crystallographic information; 3D view; checkCIF report

## Figures and Tables

**Table 1:** Hydrogen-bond geometry (Å, °)

*D*—H⋯*A*	*D*—H	H⋯*A*	*D*⋯*A*	*D*—H⋯*A*
O6—H6⋯O9^i^	0.82	1.84	2.642 (4)	165

Symmetry code: (i) 
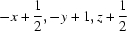
.

**Fig. 1.**
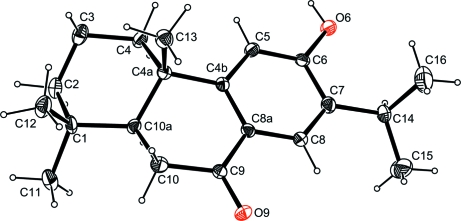
Molecular view of the title compound with the atom-labelling scheme. Displacement ellipsoids are drawn at the 30% probability level. H atoms are represented as small spheres of arbitrary radii.

**Fig. 2.**
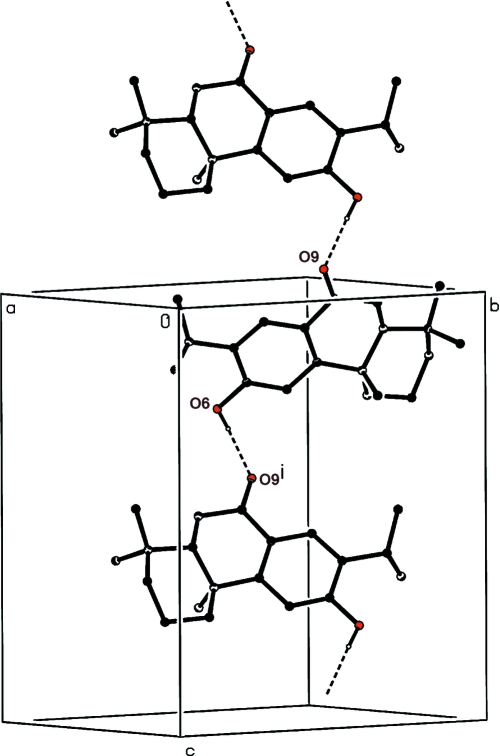
Partial packing view of the compound, showing the formation of the zig zag chain parallel to the c axis and built from O—H···O hydrogen bonds. H atoms not involved in hydrogen bonding have been omitted for clarity. [symmetry codes: (i) -x + 1/2,-y + 1, z + 1/2].

## supplementary crystallographic information

### Comment

*Juniperus oxycedrus L.* has been used in traditional folk medicine for the treatment of chronic eczema and other several skin diseases (Bouhlal *et al.*, 1988). Diterpenes are among the identified chemical constituents of this plant. They are of great interest with respect to their biological activity including antitumor, antituberculous and antimalarial effects (Iwamato *et al.*, 2003; Politi *et al.*, 2003; Ulubelen *et al.*, 1997).

The structure of the title compound is built up by three fused six-membered rings A, B and C (Fig. 1). B displays an envelope conformation with puckering parameters, Q=0.510 (4) Å, θ= 124.6 (4)° and φ= 227.1 (6)° (Cremer & Pople, 1975) whereas C has a flattened chair conformation with Q= 0.546 (4) Å, θ = 5.0 (4) and φ = 234 (5)°. A is an aromatic ring and it is perfectly planar. The molecules are linked through O—H···O hydrogen bonds involving the hydroxyl group as a donor and the ketone oxygen as an acceptor yielding a zig zag chain developing parallel to the *c* axis (Fig. 2, Table 1).

### Experimental

In order to isolate similar compounds, we have studied the chemical composition of the fruits of *Juniperus oxycedrus L*. Thus, 203 g of pulverized cones was extracted with hexane. The solvent was evaporated under reduced pressure to give 14.3 g of the crude hexanic extract which was purified on silica gel column chromatography using hexane–AcOEt (97:3) as eluent, to give crystals of Sugiol (I). All ^1^H and ^13^C NMR spectroscopic data of the isolated product were in full accord with the litterature (Bai-Ping & Isao, 1991).

### Refinement

All H atoms attached to C and O atoms were fixed geometrically and treated as riding with C—H = 0.93 Å (aromatic), 0.98 Å (methine), 0.97 Å (methylene), 0.96 Å (methyl) and O—H = 0.82 Å with *U*_iso_(H) = 1.2U_eq_ or *U*_iso_(H) = 1.5*U*_eq_(methyl, OH).

In the absence of significant anomalous scattering, the absolute configuration could not be reliably determined and then the Friedel pairs were merged and any references to the Flack parameter were removed.

### Crystal data

**Table:**

C_20_H_28_O_2_	*F*_000_ = 656
*M**_r_* = 300.42	*D*_x_ = 1.164 Mg m^−^^3^
Orthorhombic, *P*2_1_2_1_2_1_	Mo *K*α radiation λ = 0.71073 Å
Hall symbol: P 2ac 2ab	Cell parameters from 3571 reflections
*a* = 9.6060 (4) Å	θ = 2.7–32.1º
*b* = 12.6617 (6) Å	µ = 0.07 mm^−^^1^
*c* = 14.0920 (7) Å	*T* = 180 (2) K
*V* = 1713.99 (14) Å^3^	Flattened box, colorless
*Z* = 4	0.31 × 0.16 × 0.07 mm

### Data collection

**Table:**

Oxford Diffraction Xcalibur diffractometer	2003 independent reflections
Radiation source: fine-focus sealed tube	1212 reflections with *I* > 2σ(*I*)
Monochromator: graphite	*R*_int_ = 0.068
Detector resolution: 8.2632 pixels mm^-1^	θ_max_ = 26.4º
*T* = 180(2) K	θ_min_ = 2.7º
ω and φ scans	*h* = −12→12
Absorption correction: none	*k* = −15→15
13398 measured reflections	*l* = −14→17

### Refinement

**Table:**

Refinement on *F*^2^	Secondary atom site location: difference Fourier map
Least-squares matrix: full	Hydrogen site location: inferred from neighbouring sites
*R*[*F*^2^ > 2σ(*F*^2^)] = 0.046	H-atom parameters constrained
*wR*(*F*^2^) = 0.154	*w* = 1/[σ^2^(*F*_o_^2^) + (0.0842*P*)^2^] where *P* = (*F*_o_^2^ + 2*F*_c_^2^)/3
*S* = 1.05	(Δ/σ)_max_ < 0.001
2003 reflections	Δρ_max_ = 0.36 e Å^−^^3^
205 parameters	Δρ_min_ = −0.38 e Å^−^^3^
Primary atom site location: structure-invariant direct methods	Extinction correction: none

### Special details

**Table:**

Geometry. All e.s.d.'s (except the e.s.d. in the dihedral angle between two l.s. planes) are estimated using the full covariance matrix. The cell e.s.d.'s are taken into account individually in the estimation of e.s.d.'s in distances, angles and torsion angles; correlations between e.s.d.'s in cell parameters are only used when they are defined by crystal symmetry. An approximate (isotropic) treatment of cell e.s.d.'s is used for estimating e.s.d.'s involving l.s. planes.
Refinement. Refinement of F^2^ against ALL reflections. The weighted R-factor wR and goodness of fit S are based on F^2^, conventional R-factors R are based on F, with F set to zero for negative F^2^. The threshold expression of F^2^ > 2σ(F^2^) is used only for calculating R-factors(gt) etc. and is not relevant to the choice of reflections for refinement. R-factors based on F^2^ are statistically about twice as large as those based on F, and R-factors based on ALL data will be even larger.

### Fractional atomic coordinates and isotropic or equivalent isotropic displacement parameters (Å^2^)

**Table:**

	*x*	*y*	*z*	*U*_iso_*/*U*_eq_	
C1	0.0789 (4)	0.8588 (3)	0.0839 (3)	0.0291 (10)	
C2	0.1754 (4)	0.9176 (3)	0.1515 (3)	0.0339 (11)	
H2A	0.2674	0.9211	0.1234	0.041*	
H2B	0.1418	0.9894	0.1588	0.041*	
C3	0.1869 (5)	0.8673 (3)	0.2486 (3)	0.0366 (11)	
H3A	0.0966	0.8681	0.2794	0.044*	
H3B	0.2511	0.9077	0.2874	0.044*	
C4	0.2389 (4)	0.7525 (3)	0.2400 (3)	0.0296 (10)	
H4A	0.2430	0.7215	0.3029	0.036*	
H4B	0.3327	0.7531	0.2145	0.036*	
C4A	0.1464 (4)	0.6828 (3)	0.1762 (3)	0.0217 (9)	
C4B	0.2182 (4)	0.5776 (3)	0.1555 (3)	0.0202 (9)	
C5	0.3090 (4)	0.5314 (3)	0.2203 (3)	0.0252 (9)	
H5	0.3286	0.5661	0.2768	0.030*	
C6	0.3699 (4)	0.4356 (3)	0.2023 (3)	0.0255 (9)	
C7	0.3444 (4)	0.3792 (3)	0.1186 (3)	0.0296 (10)	
C8	0.2533 (4)	0.4252 (3)	0.0564 (3)	0.0244 (10)	
H8	0.2335	0.3898	0.0002	0.029*	
C8A	0.1892 (4)	0.5211 (3)	0.0724 (3)	0.0219 (9)	
C9	0.0961 (4)	0.5626 (3)	0.0010 (3)	0.0220 (9)	
C10	0.0395 (4)	0.6713 (3)	0.0137 (3)	0.0307 (10)	
H10A	−0.0545	0.6663	0.0384	0.037*	
H10B	0.0344	0.7054	−0.0479	0.037*	
C10A	0.1257 (4)	0.7408 (3)	0.0804 (3)	0.0227 (9)	
H10C	0.2186	0.7426	0.0518	0.027*	
C11	0.0945 (5)	0.9073 (3)	−0.0143 (3)	0.0455 (13)	
H11A	0.0847	0.9826	−0.0099	0.068*	
H11B	0.0239	0.8795	−0.0556	0.068*	
H11C	0.1847	0.8905	−0.0393	0.068*	
C12	−0.0741 (4)	0.8754 (3)	0.1138 (3)	0.0397 (12)	
H12A	−0.1013	0.9468	0.1006	0.060*	
H12B	−0.0836	0.8618	0.1805	0.060*	
H12C	−0.1328	0.8278	0.0790	0.060*	
C13	0.0112 (4)	0.6551 (3)	0.2292 (3)	0.0333 (11)	
H13A	−0.0494	0.6162	0.1879	0.050*	
H13B	−0.0343	0.7190	0.2490	0.050*	
H13C	0.0328	0.6130	0.2839	0.050*	
C14	0.4133 (5)	0.2730 (3)	0.1025 (3)	0.0322 (11)	
H14	0.5100	0.2792	0.1240	0.039*	
C15	0.4171 (6)	0.2384 (4)	0.0019 (4)	0.0672 (18)	
H15A	0.3239	0.2261	−0.0201	0.101*	
H15B	0.4701	0.1743	−0.0032	0.101*	
H15C	0.4598	0.2923	−0.0362	0.101*	
C16	0.3443 (6)	0.1868 (4)	0.1627 (4)	0.0614 (16)	
H16A	0.2516	0.1744	0.1399	0.092*	
H16B	0.3406	0.2093	0.2277	0.092*	
H16C	0.3976	0.1229	0.1582	0.092*	
O6	0.4598 (3)	0.3917 (2)	0.2654 (2)	0.0353 (8)	
H6	0.4641	0.4287	0.3131	0.053*	
O9	0.0639 (3)	0.5113 (2)	−0.06942 (18)	0.0304 (7)	

### Atomic displacement parameters (Å^2^)

**Table:**

	*U*^11^	*U*^22^	*U*^33^	*U*^12^	*U*^13^	*U*^23^
C1	0.033 (2)	0.018 (2)	0.036 (2)	0.0086 (18)	−0.001 (2)	0.0033 (19)
C2	0.033 (2)	0.021 (2)	0.048 (3)	0.0042 (19)	0.000 (2)	−0.002 (2)
C3	0.032 (2)	0.029 (2)	0.048 (3)	0.001 (2)	−0.008 (2)	−0.014 (2)
C4	0.032 (2)	0.027 (2)	0.029 (2)	0.0046 (19)	−0.0046 (19)	−0.0085 (19)
C4A	0.022 (2)	0.021 (2)	0.022 (2)	−0.0028 (16)	−0.0010 (18)	0.0000 (17)
C4B	0.0183 (18)	0.0196 (19)	0.023 (2)	−0.0009 (16)	0.0041 (18)	0.0026 (17)
C5	0.028 (2)	0.025 (2)	0.023 (2)	−0.0008 (18)	0.000 (2)	−0.0038 (18)
C6	0.029 (2)	0.028 (2)	0.019 (2)	0.0069 (19)	0.0023 (18)	0.0033 (19)
C7	0.032 (3)	0.025 (2)	0.032 (2)	0.0043 (19)	0.007 (2)	0.002 (2)
C8	0.028 (2)	0.023 (2)	0.022 (2)	0.0025 (19)	−0.0002 (18)	0.0010 (18)
C8A	0.026 (2)	0.0196 (19)	0.020 (2)	−0.0039 (17)	−0.0026 (19)	0.0021 (18)
C9	0.024 (2)	0.021 (2)	0.020 (2)	−0.0005 (17)	0.0018 (18)	0.0018 (18)
C10	0.033 (2)	0.032 (2)	0.027 (2)	0.0024 (19)	−0.006 (2)	−0.0011 (19)
C10A	0.021 (2)	0.024 (2)	0.023 (2)	0.0048 (16)	−0.0008 (18)	−0.0035 (18)
C11	0.059 (3)	0.029 (3)	0.049 (3)	0.008 (2)	0.001 (3)	0.011 (2)
C12	0.035 (3)	0.035 (3)	0.048 (3)	0.013 (2)	−0.009 (2)	−0.010 (2)
C13	0.030 (2)	0.033 (2)	0.037 (3)	0.0033 (19)	0.003 (2)	0.003 (2)
C14	0.036 (3)	0.025 (2)	0.035 (2)	0.010 (2)	−0.002 (2)	0.0025 (18)
C15	0.093 (5)	0.065 (4)	0.044 (3)	0.045 (4)	0.001 (3)	−0.011 (3)
C16	0.067 (4)	0.036 (3)	0.080 (4)	0.006 (3)	0.017 (3)	0.006 (3)
O6	0.0436 (18)	0.0353 (17)	0.0271 (17)	0.0196 (15)	−0.0073 (15)	−0.0031 (14)
O9	0.0393 (17)	0.0288 (15)	0.0231 (15)	0.0002 (14)	−0.0061 (14)	−0.0076 (13)

### Geometric parameters (Å, °)

**Table:**

C1—C11	1.521 (6)	C8A—C9	1.445 (5)
C1—C2	1.524 (6)	C9—O9	1.226 (4)
C1—C12	1.543 (6)	C9—C10	1.491 (5)
C1—C10A	1.561 (5)	C10—C10A	1.530 (5)
C2—C3	1.514 (6)	C10—H10A	0.9700
C2—H2A	0.9700	C10—H10B	0.9700
C2—H2B	0.9700	C10A—H10C	0.9800
C3—C4	1.542 (5)	C11—H11A	0.9600
C3—H3A	0.9700	C11—H11B	0.9600
C3—H3B	0.9700	C11—H11C	0.9600
C4—C4A	1.542 (5)	C12—H12A	0.9600
C4—H4A	0.9700	C12—H12B	0.9600
C4—H4B	0.9700	C12—H12C	0.9600
C4A—C4B	1.528 (5)	C13—H13A	0.9600
C4A—C13	1.539 (5)	C13—H13B	0.9600
C4A—C10A	1.550 (5)	C13—H13C	0.9600
C4B—C5	1.391 (5)	C14—C15	1.485 (6)
C4B—C8A	1.401 (5)	C14—C16	1.532 (6)
C5—C6	1.370 (5)	C14—H14	0.9800
C5—H5	0.9300	C15—H15A	0.9600
C6—O6	1.359 (4)	C15—H15B	0.9600
C6—C7	1.401 (5)	C15—H15C	0.9600
C7—C8	1.369 (5)	C16—H16A	0.9600
C7—C14	1.517 (5)	C16—H16B	0.9600
C8—C8A	1.380 (5)	C16—H16C	0.9600
C8—H8	0.9300	O6—H6	0.8200
			
C11—C1—C2	108.1 (4)	C8A—C9—C10	118.6 (3)
C11—C1—C12	106.7 (4)	C9—C10—C10A	114.0 (3)
C2—C1—C12	110.0 (3)	C9—C10—H10A	108.7
C11—C1—C10A	109.3 (3)	C10A—C10—H10A	108.7
C2—C1—C10A	108.2 (3)	C9—C10—H10B	108.7
C12—C1—C10A	114.4 (3)	C10A—C10—H10B	108.7
C3—C2—C1	113.9 (3)	H10A—C10—H10B	107.6
C3—C2—H2A	108.8	C10—C10A—C4A	109.4 (3)
C1—C2—H2A	108.8	C10—C10A—C1	114.4 (3)
C3—C2—H2B	108.8	C4A—C10A—C1	117.6 (3)
C1—C2—H2B	108.8	C10—C10A—H10C	104.6
H2A—C2—H2B	107.7	C4A—C10A—H10C	104.6
C2—C3—C4	110.4 (3)	C1—C10A—H10C	104.6
C2—C3—H3A	109.6	C1—C11—H11A	109.5
C4—C3—H3A	109.6	C1—C11—H11B	109.5
C2—C3—H3B	109.6	H11A—C11—H11B	109.5
C4—C3—H3B	109.6	C1—C11—H11C	109.5
H3A—C3—H3B	108.1	H11A—C11—H11C	109.5
C3—C4—C4A	113.5 (3)	H11B—C11—H11C	109.5
C3—C4—H4A	108.9	C1—C12—H12A	109.5
C4A—C4—H4A	108.9	C1—C12—H12B	109.5
C3—C4—H4B	108.9	H12A—C12—H12B	109.5
C4A—C4—H4B	108.9	C1—C12—H12C	109.5
H4A—C4—H4B	107.7	H12A—C12—H12C	109.5
C4B—C4A—C13	106.0 (3)	H12B—C12—H12C	109.5
C4B—C4A—C4	110.5 (3)	C4A—C13—H13A	109.5
C13—C4A—C4	109.5 (3)	C4A—C13—H13B	109.5
C4B—C4A—C10A	107.8 (3)	H13A—C13—H13B	109.5
C13—C4A—C10A	115.0 (3)	C4A—C13—H13C	109.5
C4—C4A—C10A	108.1 (3)	H13A—C13—H13C	109.5
C5—C4B—C8A	117.3 (3)	H13B—C13—H13C	109.5
C5—C4B—C4A	121.6 (3)	C15—C14—C7	114.6 (4)
C8A—C4B—C4A	121.0 (3)	C15—C14—C16	109.2 (4)
C6—C5—C4B	121.3 (4)	C7—C14—C16	111.0 (4)
C6—C5—H5	119.4	C15—C14—H14	107.2
C4B—C5—H5	119.4	C7—C14—H14	107.2
O6—C6—C5	120.9 (4)	C16—C14—H14	107.2
O6—C6—C7	117.0 (3)	C14—C15—H15A	109.5
C5—C6—C7	122.2 (4)	C14—C15—H15B	109.5
C8—C7—C6	115.8 (3)	H15A—C15—H15B	109.5
C8—C7—C14	124.1 (4)	C14—C15—H15C	109.5
C6—C7—C14	120.1 (4)	H15A—C15—H15C	109.5
C7—C8—C8A	123.7 (4)	H15B—C15—H15C	109.5
C7—C8—H8	118.2	C14—C16—H16A	109.5
C8A—C8—H8	118.2	C14—C16—H16B	109.5
C8—C8A—C4B	119.8 (4)	H16A—C16—H16B	109.5
C8—C8A—C9	118.9 (3)	C14—C16—H16C	109.5
C4B—C8A—C9	121.3 (3)	H16A—C16—H16C	109.5
O9—C9—C8A	121.7 (3)	H16B—C16—H16C	109.5
O9—C9—C10	119.6 (4)	C6—O6—H6	109.5

### Hydrogen-bond geometry (Å, °)

**Table:**

*D*—H···*A*	*D*—H	H···*A*	*D*···*A*	*D*—H···*A*
O6—H6···O9^i^	0.82	1.84	2.642 (4)	165

Symmetry codes: (i) −*x*+1/2, −*y*+1, *z*+1/2.
